# Correlations between metabolism and structural elements of the alicyclic fentanyl analogs cyclopropyl fentanyl, cyclobutyl fentanyl, cyclopentyl fentanyl, cyclohexyl fentanyl and 2,2,3,3-tetramethylcyclopropyl fentanyl studied by human hepatocytes and LC-QTOF-MS

**DOI:** 10.1007/s00204-018-2330-9

**Published:** 2018-10-25

**Authors:** Anna Åstrand, Amanda Töreskog, Shimpei Watanabe, Robert Kronstrand, Henrik Gréen, Svante Vikingsson

**Affiliations:** 1 Department of Medical and Health Sciences, Linköping University, Linköping, Sweden; 2 Department of Forensic Genetics and Forensic Toxicology, National Board of Forensic Medicine, Linköping, Sweden

**Keywords:** Fentanyl analogs, Metabolism, Human hepatocytes, LC-QTOF-MS

## Abstract

Recently, a number of fentanyl analogs have been implicated in overdose deaths in Europe and in the US. So far, little is known of the molecular behavior of the structurally related subgroup; the alicyclic fentanyls. In this study, reference standards of cyclopropyl, cyclobutyl, cyclopentyl, cyclohexyl, and 2,2,3,3-tetramethylcyclopropyl fentanyl (TMCPF) at a final concentration of 5 µM were incubated with cryopreserved human hepatocytes (1 × 10^6^ cells/mL) for 0, 1, 3 and 5 h. The metabolites formed were identified by liquid chromatography–quadrupole time-of-flight mass spectrometry analysis. The most abundant biotransformation found was *N*-dealkylation (formation of normetabolites) and oxidation of the alicyclic rings. As ring size increased, the significance of *N*-dealkylation decreased in favor of alicyclic ring oxidation. An example of this was cyclopropyl fentanyl, with a three-carbon ring, whose normetabolite covered 82% of the total metabolic peak area and no oxidation of the alicyclic ring was observed. In contrast, TMCPF, with a seven-carbon ring structure, rendered as much as 85% of its metabolites oxidized on the alicyclic ring. Other biotransformations found included oxidation of the piperidine ethyl moiety and/or the phenethyl substructure, glucuronidation as well as amide hydrolysis to form metabolites identical to despropionyl fentanyl. Taken together, this study provides a base for understanding the metabolism of a number of structurally related fentanyl analogs formed upon intake.

**Electronic supplementary material:**

The online version of this article (10.1007/s00204-018-2330-9) contains supplementary material, which is available to authorized users.

## Introduction

Analogs of fentanyl are a widespread and lethal group of new psychoactive substances (NPS). Fentanyl analogs are reported to be implicated in numerous opioid overdose cases both in Europe (EMCDDA 2017a, b, c) and North America (O’Donnell et al. 2017).

Recently, Sweden has seen a number of fentanyl analog-related fatalities that included substances such as acryl, furanyl and cyclopropyl fentanyl (EMCDDA 2018; Guerrieri et al. 2017a, b). In particular, cyclopropyl fentanyl has been extensively used. Cyclopropyl fentanyl was over the time period June–October 2017 associated with 59 deaths in Sweden and one in Norway (EMCDDA 2018). In the United States, cyclopropyl fentanyl has been associated with at least 115 deaths since May 2017 (Drug Enforcement Administration 2018a).

In addition to cyclopropyl fentanyl, two other alicyclic fentanyl analogs have also been identified in Sweden. Cyclopentyl fentanyl was analytically confirmed in tablets (Helander et al. 2017) and 2,2,3,3-tetramethylcyclopropyl fentanyl (TMCPF) was identified by the Swedish National Forensic Center. The US Drug Enforcement Administration also reported findings of cyclopentyl fentanyl in the US (Drug Enforcement Administration 2018b).

It is imperative for forensic toxicology laboratories to analytically confirm intakes of fentanyl and fentanyl analogs, including the alicyclic fentanyl analogs, to establish cause of death as well as illicit drug use. In addition, identifying metabolites is essential to understand the pharmacokinetics and toxicology these drugs. By comparing the metabolism of closely related compounds, the effects of structural elements on metabolism can be studied. Such information might be beneficial in the study of future analogs.

Fentanyl is a well-known potent µ-receptor agonist which mediates respiratory depression, the common cause for fatalities associated with opioid use (Rang 2007). Similarly, both cyclopropyl and cyclopentyl fentanyl were reported as µ-receptor agonists (Drug Enforcement Administration 2018a, b).

The metabolism of fentanyl and a few fentanyl analogs was studied using urine samples and in vitro model systems including human liver microsomes and human hepatocytes. Multi-donor human hepatocytes have proven to be a useful system to study the metabolism of fentanyls and identified metabolites correlate well with those found in authentic urine samples (Watanabe et al. 2017).

Goromaru et al. showed that 26–55% of fentanyl was excreted as norfentanyl in urine during the first 12 h after administration (Goromaru et al. 1984). Hydroxylations on the propionyl side chain of both fentanyl and norfentanyl were reported as minor metabolites in urine samples (Goromaru et al. 1984; Mahlke et al. 2014).

Many fentanyl analogs such as acetyl, acryl and 4-chloro-isobutyryl fentanyl show a similar metabolism dominated by *N*-dealkylation to form the normetabolite. Oxidation reactions on the phenethyl substructure (Kanamori et al. 2018; Watanabe et al. 2017) are also observed. However, some fentanyl analogs undergo different biotransformations. For example, the metabolism of butyr fentanyl also includes oxidation on the butyr side chain (Steuer et al. 2017). Moreover, for furanyl fentanyl, biotransformations such as oxidation on the furanyl ring and amide hydrolysis to form the desalkyl metabolite occur (Watanabe et al. 2017).

To study similarities and differences in the metabolism of closely related fentanyl analogs, we incubated five alicyclic fentanyls; cyclopropyl, cyclobutyl, cyclopentyl, cyclohexyl fentanyl and TMCPF, that differ only in the number of carbon present in the alicyclic ring, with cryopreserved hepatocytes and analyzed the produced metabolites by liquid chromatography–quadrupole time-of-flight mass spectrometry (LC-QTOF-MS). Major metabolites of all five analogs were identified and compared to investigate the correlation between the chemical structure and formed metabolites.

## Materials and methods

In this study, metabolites of cyclopropyl, cyclobutyl, cyclopentyl, cyclohexyl fentanyl and TMCPF were produced by incubation with cryopreserved human hepatocytes for up to 5 h. Formed metabolites were analyzed using LC-QTOF-MS and identified by their MSMS spectra and accurate mass.

### Chemicals and reagents

Reference materials (cyclopropyl, cyclobutyl, cyclopentyl, cyclohexyl, and 2,2,3,3-tetramethylcyclopropyl fentanyl) were purchased from Cayman Chemicals (Ann Arbor, MI, USA). Diclofenac was obtained from Sigma-Aldrich (Stockholm, Sweden). Solvents and formic acid for the LC-QTOF-MS analysis were obtained from ThermoFisher Scientific (Gothenburg, Sweden). Ammonium formate (Fluka) was purchased from Sigma-Aldrich (Stockholm, Sweden). Cryopreserved human hepatocytes (LiverPool, 10 donor pool) and inVitro Gro HT thawing medium were obtained from Bioreclamation IVT (Brussels, Belgium). Williams E medium, l-glutamine and HEPES buffer were purchased from ThermoFisher Scientific.

### Metabolite production by incubation with human hepatocyte and sample preparation

For metabolite production, the drugs were incubated in duplicate with human hepatocytes for 0, 1, 3 and 5 h using a slightly modified version of the protocol used by Watanabe et al. (2017). In brief, the hepatocytes were thawed at 37 °C and diluted in 48 mL thawing medium (in vitro Gro HT). Cells were pelleted by 5 min centrifugation at 60–100 g at room temperature and washed once in 50 mL incubation medium (Williams E medium, supplemented with 2 mM l-glutamin and 20 mM HEPES). Cell concentration was adjusted to 2 × 10^6^ viable cells/mL, as determined by the Trypan blue exclusion method, in fresh medium.

Working solutions of the drugs at 10 µM in incubation medium were prepared. 50 µL of the working solution was mixed with 50 µL cell suspension for a final substrate concentration of 5 µM and 1 × 10^6^ cells/mL in 96-well plates. The cells were incubated in the presence of the drugs for 1, 3 or 5 h at 37 °C, 5% CO_2_. The reactions were terminated by the addition of 100 µL ice-cold acetonitrile and extracts were frozen at − 20 °C until analysis (minimum of 10 min). For 0 h control samples, acetonitrile was added to the 96-well plate prior to the cell suspension. Diclofenac (5 µM) was used as a positive control. Negative (without drug) and degradation (without cells present) control samples were incubated for 5 h. Immediately before analysis by LC-QTOF-MS, the extracts were centrifuged at 1100*g* for 15 min at + 4 °C and 100 µL of the supernatant was transferred to an injection plate. In the following LC-QTOF-MS analysis, 1 µL of the supernatant was injected.

### LC-QTOF-MS analysis

The chromatographic system comprised an Agilent 1290 Infinity ultra-high performance liquid chromatography system (Kista, Sweden) and an Agilent 6550 iFunnel QTOF. Chromatographic separation was achieved on an Acquity HSS T3 column (150 × 2.1 mm, 1.8 µm) (Waters, Sollentuna, Sweden). Mobile phases used were 0.05% formic acid and 10 mM ammonium formate in water (A) and 0.05% formic acid in acetonitrile (B). For TMCPF, cyclopropyl, and cyclobutyl fentanyl a gradient changing the mobile phase B from 1 to 40% was used while for cyclopentyl and cyclohexyl fentanyl a gradient from 25 to 65% B was used. The gradient program used was consistent for both gradients holding 1% B for 0.6 min followed by a ramp up to the lower end (1 and 25% B, respectively) at 0.7 min. Using a linear gradient, the high end of the gradient (40 and 65%, respectively) was reached after 13 min. This was followed by a ramp up to 95% B at 15 min which was held until 18 min before returning to 1% B which was held for 1 min. The gradients were selected to ensure retention times of the parent drugs within 8–13 min.

The QTOF was operated in positive electrospray (gas temperature, 150 °C; gas flow, 18 L/min; nebulizer, 50 psig; sheath gas temperature, 375 °C; sheath gas flow, 11 L/min). MS data were obtained using Data Dependent Auto MS/MS [scan rate, 6 spectra/s (MS) and 10 spectra/s (MSMS); scan range 100–950 *m*/*z* (MS) and 50–950 *m*/*z* (MSMS); precursor intensity threshold, 5000 counts; precursor number per cycle, 5 within 200–800 *m*/*z*; fragmentor voltage, 380 V; collision energy, 3 eV at 0 *m*/*z* ramped up by 8 eV per 100 *m*/*z*].

### Data analysis

Data analysis was performed using MassHunter Qualitative Analysis. Libraries containing potential metabolite formulae for mono-, di- and trihydroxylations, carbonylation, carboxylation, methylation, dihydrodiol formation, amide hydrolysis, glucuronidation, sulfate conjugation and *N*-dealkylation to form normetabolites as well as combinations thereof were compiled and used to search for metabolites. In this study, the top ten most abundant metabolites for each drug, based on the peak area in the 5 h samples were reported. Unless saturated, only peaks with a mass error < 5 ppm were considered.

## Results

The ten most abundant metabolites of cyclobutyl, cyclopentyl, cyclohexyl fentanyl, and TMPCF produced by incubation with human hepatocytes, were identified. For cyclopropyl fentanyl, only seven metabolites were detected. Observed biotransformations included loss of the phenethyl substructure by *N*-dealkylation (formation of normetabolites) and loss of the alicyclic ring by amide hydrolysis (formation of a metabolite identical to despropionyl fentanyl) as well as hydroxylations and other oxidation reactions on the alicyclic rings, the piperidine ethyl moiety (including two *N*-oxides) and on the phenethyl substructure. Two glucuronidated metabolites of cyclopropyl fentanyl and TMCPF were also found. The identity of all metabolites with retention times, formulae, mass error, peak areas and diagnostic fragment ions is shown as Supplementary Tables S1–S5.

### The metabolic profile of cyclopropyl fentanyl

**Fig. 1:**
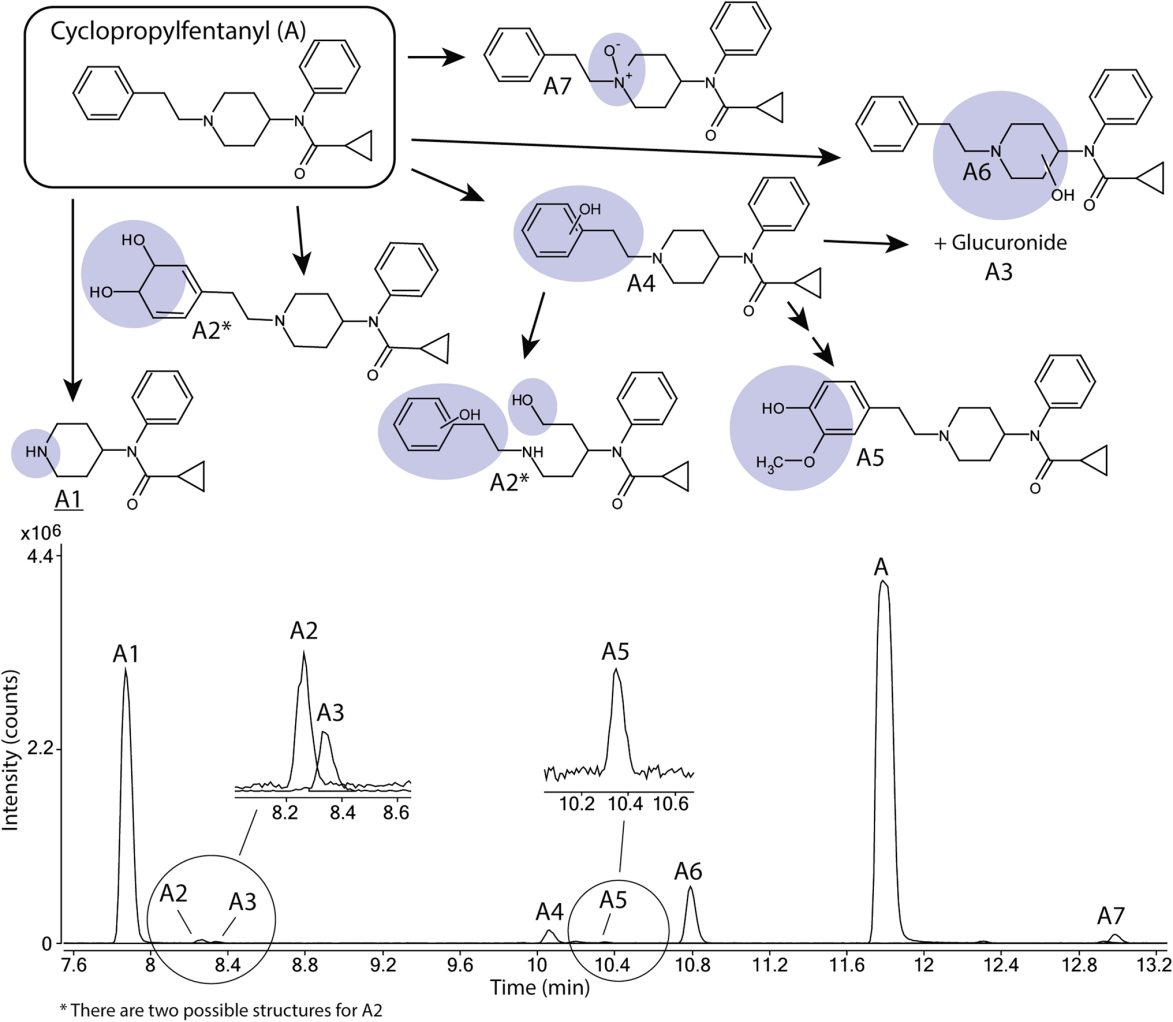
Proposed metabolic pathway of cyclopropylfentanyl (A, top) with the modifications highlighted in blue and major metabolites underlined. Markush structures are used to indicate when several positions of the modifications are possible with the exceptions of A2 and A5 where modifications are drawn as one possible isomer. Extracted ion chromatograms of cyclopropylfentanyl and its corresponding metabolites formed after 5 h of incubation with human hepatocytes are also depicted (bottom) with close-ups of the minor metabolites A2, A3 and A5. (Color figure online)

Seven metabolites (A1–A7) of cyclopropyl fentanyl (A) were identified as shown in Fig. 1 and Supplemental Figure S1. The major metabolite with a peak area of 79% of total metabolite area after 5 h was the normetabolite (A1). The second most intense metabolite was A6 (14% of total metabolite area), which underwent monohydroxylation at the piperidine ethyl moiety.

Minor metabolites (0.4–3.3% of total metabolite area) include the metabolite A4, monohydroxylated on the phenethyl substructure, a hydroxy methoxy metabolite (A5), one dihydrodiol (alternatively a ring-opened dihydroxylated metabolite) (A2) and one *N*-oxide (A7). One glucuronidated metabolite (A3) was also observed. Notably, no metabolites oxidated on the cyclopropyl ring or formed by amide hydrolysis were detected.

### The metabolic profile of cyclobutyl fentanyl

**Fig. 2:**
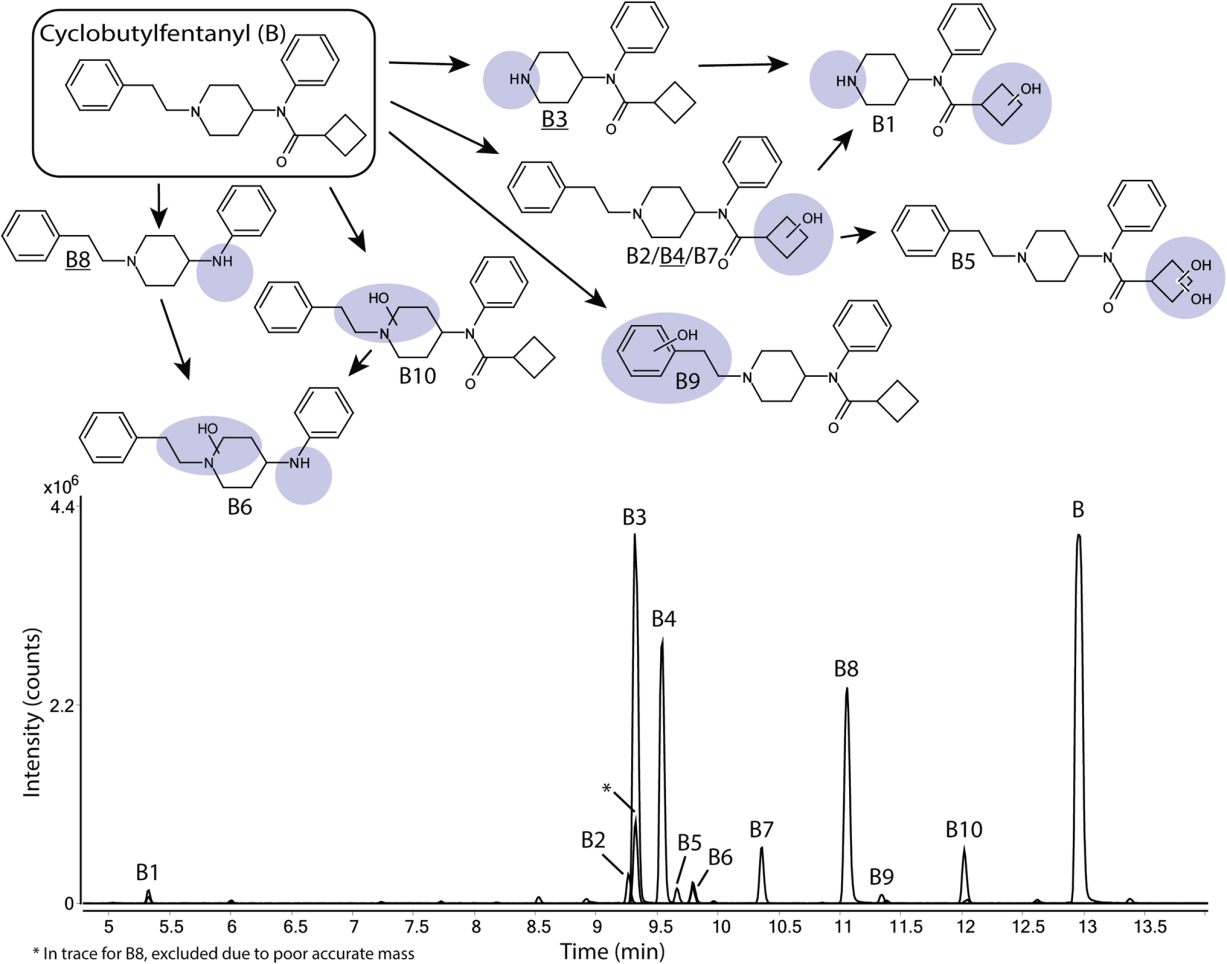
Proposed metabolic pathway of cyclobutylfentanyl (B, top) with the modifications highlighted in blue and major metabolites underlined. Markush structures are used to indicate when several positions of the modifications are possible. Extracted ion chromatograms of cyclobutylfentanyl and its corresponding metabolites formed after 5 h of incubation with human hepatocytes are also depicted (bottom). (Color figure online)

In Fig. 2 and Supplemental Figure S2, ten metabolites (B1–B10) of cyclobutyl fentanyl (B) are depicted. The normetabolite (B3), a hydroxy metabolite with the modification on the cyclobutyl ring (B4) and metabolite B8 are all major metabolites of cyclobutyl fentanyl covering 37%, 24% and 23%, respectively, of the total metabolite area after 5 h. B8 was formed by amide hydrolysis to form a dealkylated metabolite identical to despropionyl fentanyl.

Minor metabolites (0.8–4.6% of total metabolite area) include four additional monohydroxylated metabolites (B2, B7, B9 and B10). B2 and B7 are hydroxylated on the cyclobutyl ring, B9 was hydroxylated on the phenethyl substructure and B10 was hydroxylated on the piperidine ethyl moiety. Other metabolites include B5, a dihydroxylated metabolite modified on the cyclobutyl ring, a hydroxylated version of B8 (B6) and a hydroxylated normetabolite (B1).

### The metabolic profile of cyclopentyl fentanyl

**Fig. 3:**
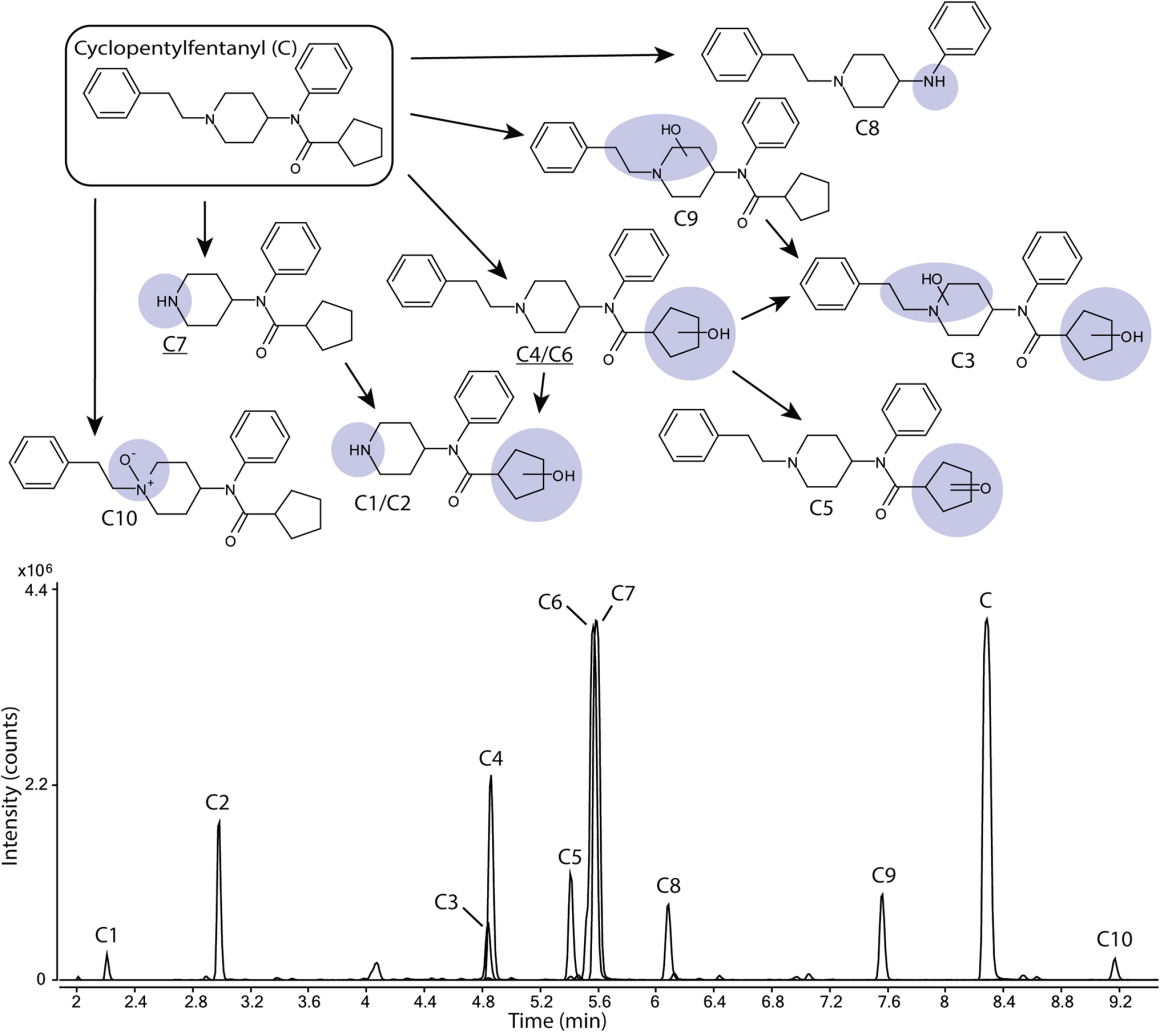
Proposed metabolic pathway of cyclopentylfentanyl (C, top) with the modifications highlighted in blue and major metabolites underlined. Markush structures are used to indicate when several positions of the modifications are possible. Extracted ion chromatograms of cyclopentylfentanyl and its corresponding metabolites formed after 5 h of incubation with human hepatocytes are also depicted (bottom). (Color figure online)

Ten metabolites (C1–C10) of cyclopentyl fentanyl (C) were identified and shown in Fig. 3 and Supplementary Figure S3. Major metabolites of cyclopentyl fentanyl were the two monohydroxylated metabolites C6 and C4 as well as the normetabolite (C7). Both C6 and C4 are hydroxylated on the cyclopentyl ring, covering 31% and 11%, respectively, of the total metabolite area after 5 h. The normetabolite C7 covers 29% of the total metabolite area.

Minor metabolites (1.0–7.6% of total metabolite area) include one additional monohydroxylated metabolite (C9), one dihydroxylated (C3) metabolite and one carbonylated (C5) metabolite as well as two hydroxylated normetabolites (C1and C2), the amide hydrolysis product (C8) and an *N*-oxide (C10).

### The metabolic profile of cyclohexyl fentanyl

**Fig. 4:**
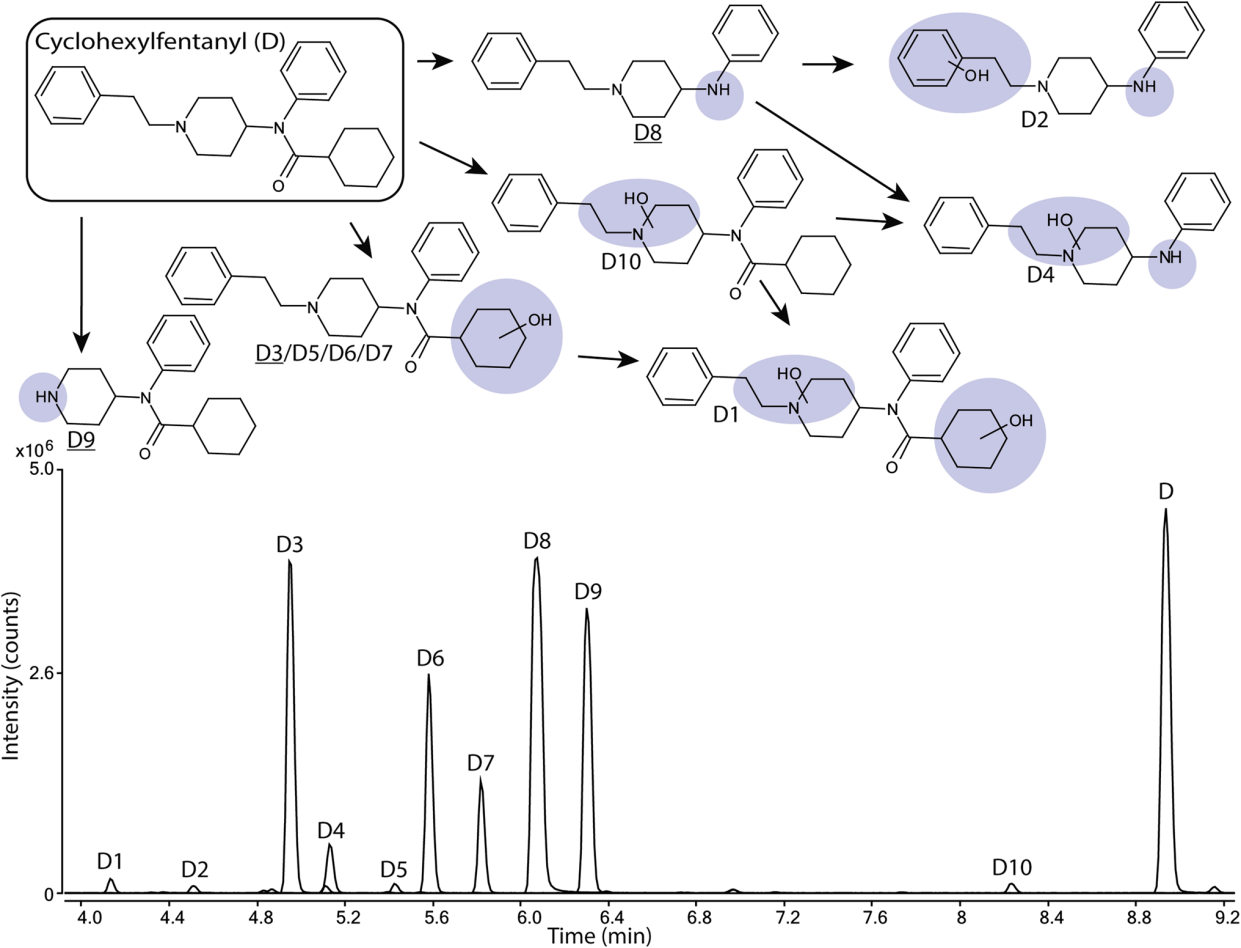
Proposed metabolic pathway of cyclohexylfentanyl (D, top) with the modifications highlighted in blue and major metabolites underlined. Markush structures are used to indicate when several positions of the modifications are possible. Extracted ion chromatograms of cyclohexylfentanyl and its corresponding metabolites formed after 5 h of incubation with human hepatocytes are also depicted (bottom). (Color figure online)

Ten metabolites (D1–D10) of cyclohexyl fentanyl (D) were identified and are shown in Fig. 4 and Supplemental Figure S4. The amide hydrolysis product (D8) was the most abundant metabolite with 32% of the total metabolite area after 5 h. Other major metabolites included the two monohydroxylated metabolites D3 and D6, both hydroxylated at the cyclohexyl ring (22 and 13% of total metabolite area, respectively) as well as the normetabolite D9 at 21% of total metabolite area.

Minor metabolites (0.5–6.6% of total metabolite area) included three monohydroxylated metabolites (D5, D7 and D10) and one dihydroxylated metabolite (D1) as well as two hydroxylated amide hydrolysis products (D2 and D4). D5 and D7 are hydroxylated at the cyclohexyl ring, whereas D10 is hydroxylated at the piperidine ethyl moiety. D1 was hydroxylated on both the piperidine ethyl moiety and cyclohexyl ring.

### The metabolic profile of TMCPF

**Fig. 5:**
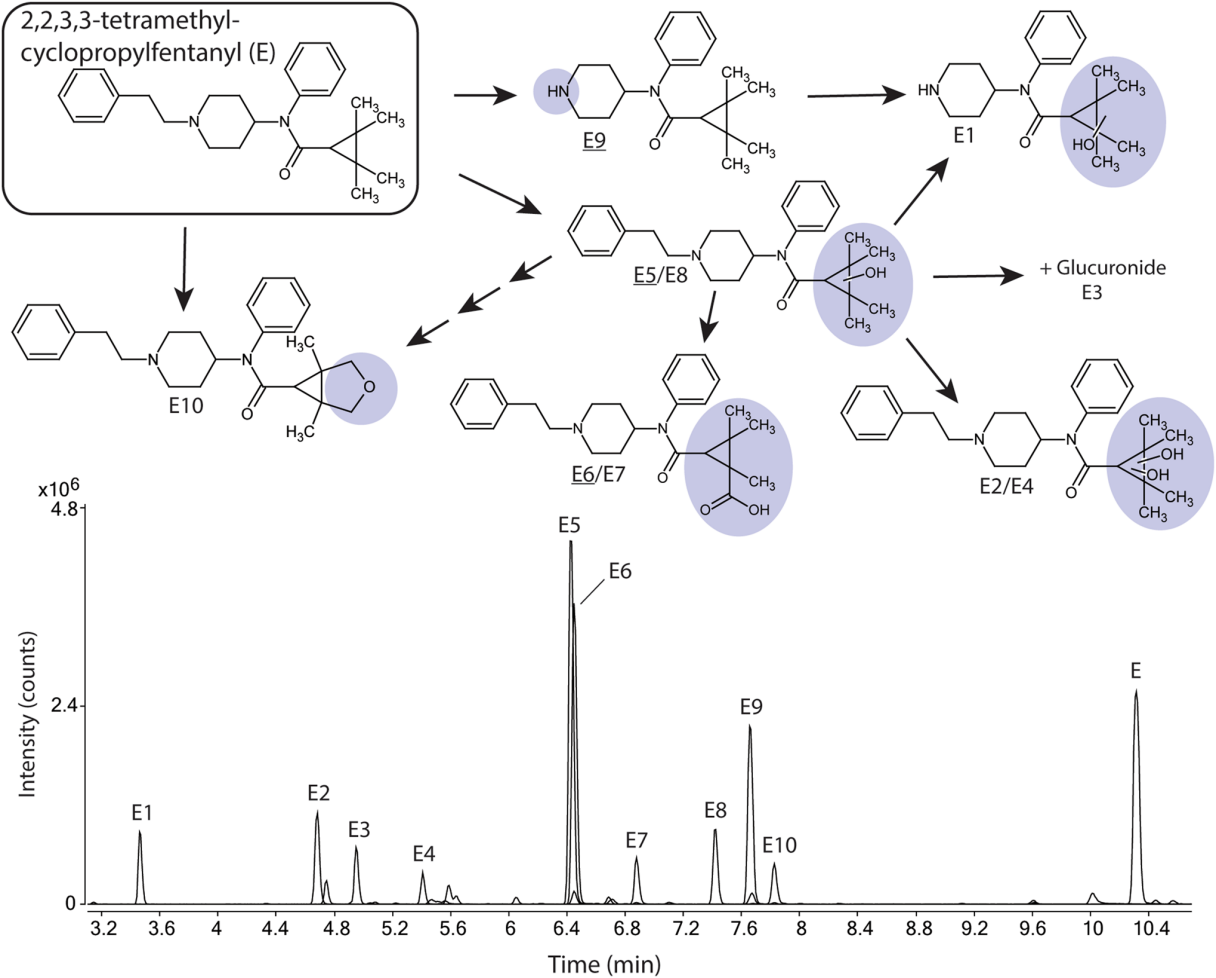
Proposed metabolic pathway of 2,2,3,3-tetramethylcyclopropylfentanyl (TMCPF, E, top) with the modifications highlighted in blue and major metabolites underlined. Markush structures are used to indicate when several positions of the modifications are possible with the exceptions of E6, E7 and E10 where modifications are drawn as one possible isomer. Extracted ion chromatograms of TMCPF and its corresponding metabolites formed after 5 h of incubation with human hepatocytes are also depicted (bottom). (Color figure online)

Ten metabolites (E1–E10) of TMCPF (E) were identified (Fig. 5; Supplemental Figure S5). The most intense metabolite was E5 with 34% of total metabolite area. E5 is monohydroxylated at the tetramethylcyclopropyl (TMCP) ring. The other major metabolite was E6 covering 19% of total metabolite area and for which the corresponding MSMS spectra are consistent with a carboxylic acid functionality on the TMCP ring. The carboxylic acid group was also identified for the minor metabolite E7 (see fragment analysis below).

Other minor metabolites (2.0–15% of total metabolite area) included one monohydroxylated metabolite (E8) as well as two dihydroxylated metabolites (E2, E4). All three were hydroxylated on the TMCP ring. Dealkylation to form the normetabolite without (E9) and in combination with monohydroxylation (E1) were also observed. TMCPF is one of two alicyclic fentanyl analogs that formed a glucuronide (E3). Also detected is a dihydroxylated metabolite (TMCP ring) that was further internally dehydrated (E10), see Supplemental Figure S5.

### Different types of metabolites

**Fig. 6:**
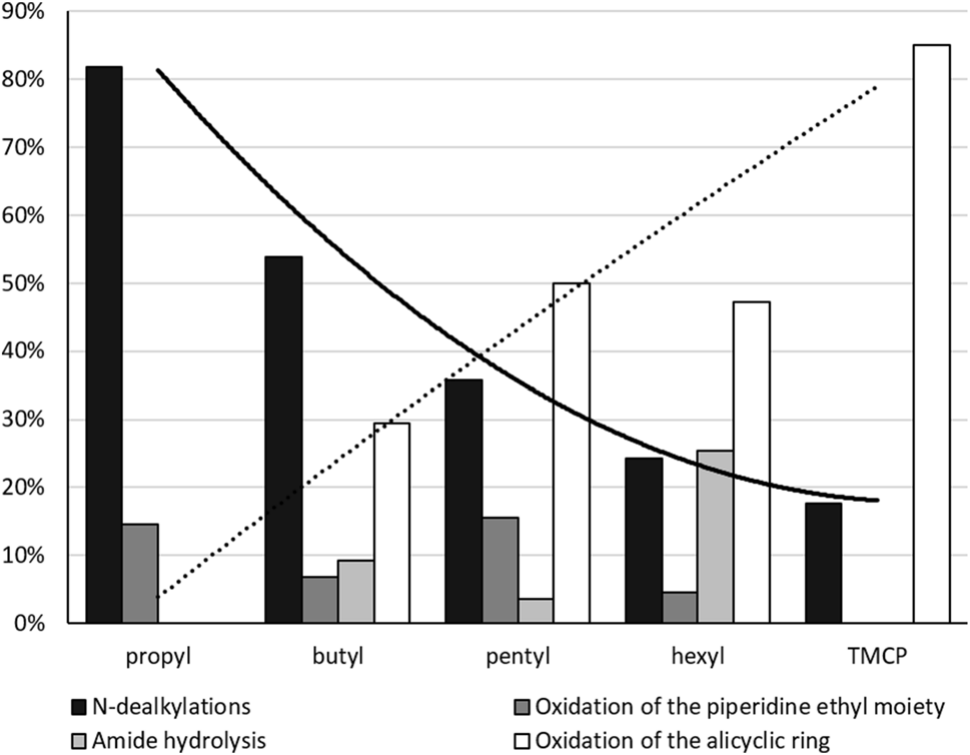
Percentages, by peak area, of normetabolites (black), metabolites oxidized on the piperidine moiety (dark gray), amide hydrolysis products (light gray) and metabolites oxidized on the aliphatic ring (white) relative to the total area of all metabolites after 1 h incubation for each fentanyl analog. Metabolites belonging to more than one group, due to multiple biotransformations or ambiguity in structure assignment, were counted in both categories. The curves illustrate the increasing/decreasing trends of metabolites oxidized on the aliphatic ring (dashed) and normetabolites (solid), as the ring sizes increases

To investigate a possible relationship between parental structure and the identity and abundance of the metabolites formed, the relative area after 1 h of incubation of four different types of metabolites (normetabolites, metabolites oxidized on the piperidine ethyl moiety, amide hydrolysis products and metabolites oxidized on the aliphatic ring) was depicted in Fig. 6. Metabolites belonging to more than one category, due to multiple biotransformations or ambiguity in structure assignment, were counted in both categories. The area of phase I metabolites oxidized on the phenethyl substructure and the phase II glucuronides were each below 5% of the total metabolite area for the respective analogs and were therefore not included in Fig. 6.

## Discussion

Using drug incubation with multi-donor human hepatocytes to model the metabolism under controlled conditions allowed for comparison of the metabolic profiles of the five drugs: cyclopropyl, cyclobutyl, cyclopentyl, cyclohexyl fentanyl and TMCPF.

### Structure elucidation of alicyclic fentanyl metabolites formed after incubation with human hepatocytes

Structure elucidation of metabolites was performed by interpretation of their respective MSMS spectra. By comparing observed fragment ions of the metabolites to those of the parent drugs, biotransformations could be assigned to specific part of the molecules. MSMS spectra of parent drugs and all metabolites as well as their respective assigned structures are shown in Supplementary Figure S1–S5.

**Fig. 7:**
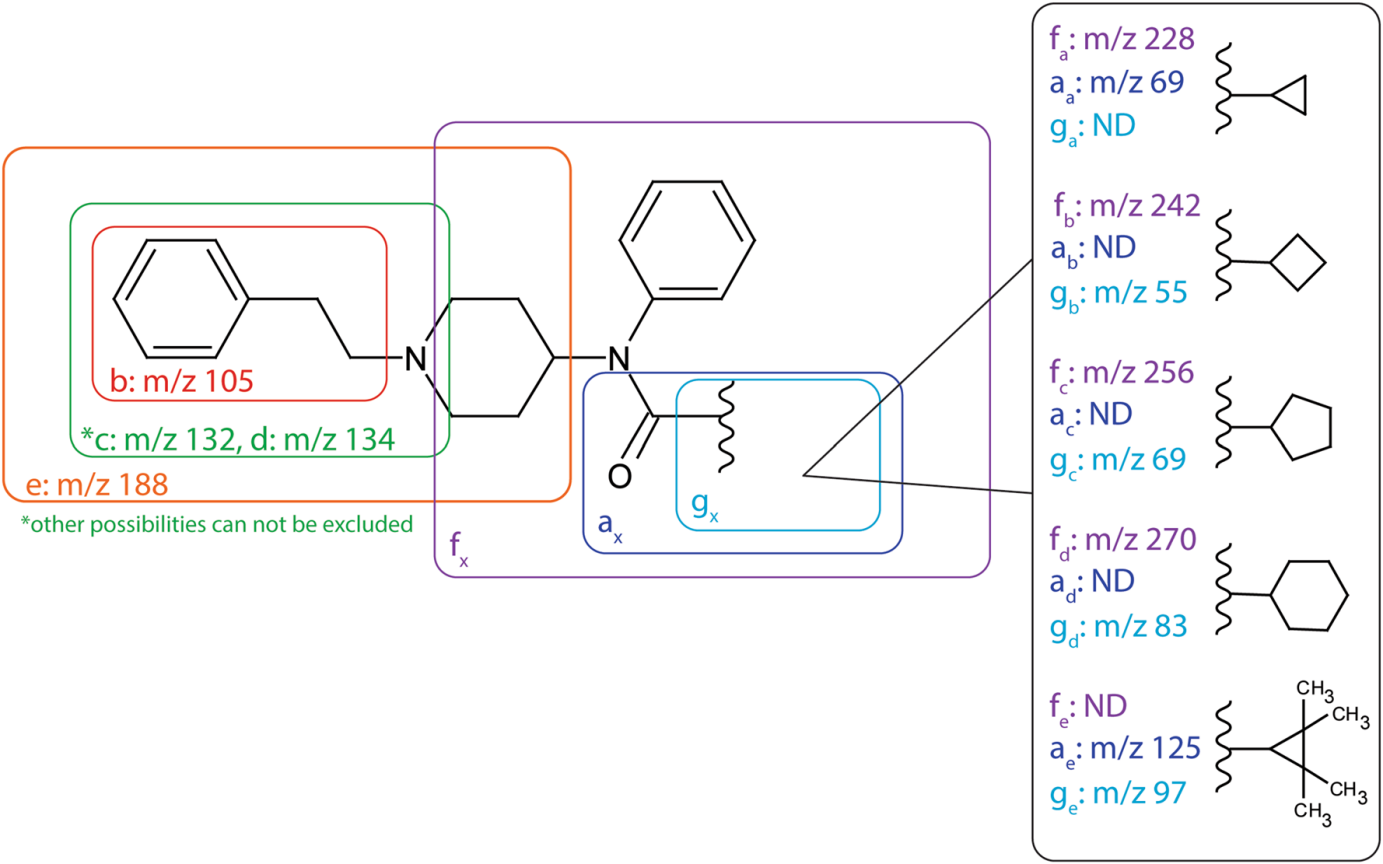
MSMS fragmentation of the alicyclic fentanyl analogs. Diagnostic fragment ions are labeled a–g, linked to their respective substructure and displayed with their corresponding *m*/*z* values. Fragments for which the *m*/*z* vary with the alicyclic ring have been marked with a subscript X and where applicable the *m*/*z* is given for each alicyclic fentanyl analog. (Color figure online)

The alicyclic fentanyl analogs fragmented mainly by bond cleavage at preferred sites. Seven fragment ions (labeled a_x_, b, c, d, e, f_x_ and g_x_) were found to be characteristic and among them four fragment ions (b, c, d and e) were identical for all five substances (Fig. 7). Fragments c, d and c + CH_2_ could only be formed by cleaving the piperidine moiety at multiple locations, thus allowing multiple assignments of the exact structure of these fragments. Similarly, for fragment f, both a ring opening of the piperidine moiety and a reduction of ring size were possible. Suggested structures for fragment ions not shown in Fig. 7 are included in Supplemental Figures S1–S5.

### Metabolite structure elucidation of common biotransformations for the five alicyclic fentanyls

In general, mono and dihydroxylated metabolites were identified by fragments b, e, a and/or g (Fig. 7). The additional mass of one oxygen or the loss of two hydrogens (indicative of water loss after hydroxylation) implies a hydroxylated phenethyl substructure (fragment b + O), piperidine ethyl moiety (fragment e + O) and/or alicyclic ring (fragment a + O/g + O). Similarly, the addition of two oxygens, addition of one oxygen and loss of two hydrogens or the loss of four hydrogens indicated a dihydroxylated moiety while a fragment with the same mass as for the parent indicated an unmodified moiety. Glucuronidated metabolites were identified in the same way as their respective phase I metabolites in combination with their precursor mass.

The normetabolites were mainly identified by fragments a and/or g as well as the presence of the piperidine cation (*m*/*z* 84). Hydroxylated normetabolites were identified similarly as monohydroxylated metabolites.

The amide hydrolysis product identical to despropionyl fentanyl was identified by precursor mass and fragments b and e. Monohydroxylated amide hydrolysis products were identified similarly as monohydroxylated metabolites.

The structure elucidation of other metabolites as well as metabolites with ambiguous spectra is discussed for the different parent drugs below.

### Biotransformations of cyclopropyl fentanyl metabolites

All spectra of cyclopropyl fentanyl and metabolites are found in Supplemental Figure S1. The normetabolite (A1), the two monohydroxylated metabolites (A4 and A6) as well as the glucuronide (A3) were identified as described above.

A2 could be either a dihydrodiol at the phenethyl substructure or contain a hydroxylated and ring-opened piperidine moiety in addition to a monohydroxylated phenethyl substructure, see Supplemental Figure S1. The water loss fragment e + O is consistent with both possibilities as is fragment a indicating an unmodified cyclopropyl ring. Interestingly, no fragment b was observed.

The dihydroxylated and methylated metabolite A5 is identified by the addition of CH_2_O_2_ to fragments b, d and e and an unmodified fragment a, indicating that all biotransformations were on the phenethyl substructure. The methylation is probably catalyzed by catechol-*O*-methyltransferase (Guldberg and Marsden 1975) as described for other fentanyl analogs (Watanabe et al. 2017). Methylation in the meta-position is most likely as this has been shown to be the preferred site of methylation for other drugs (Guldberg and Marsden 1975).

Metabolite A7 is most likely an *N*-oxide modified on the piperidine moiety nitrogen as indicated by unmodified fragments b, a and f. A7 elutes after cyclopropylfentanyl which is consistent with *N*-oxide metabolites (Cashman et al. 1992; Watanabe et al. 2017).

### Cyclobutyl fentanyl

All spectra of cyclobutyl fentanyl and metabolites are found in Supplemental Figure S2. All metabolites except the monohydroxylated metabolite B7 could be elucidated as described above.

The spectra of B7 contained unmodified fragments b and e which in combination with fragment ion *m*/*z* 281.1993, corresponding to the amide hydrolysis product. Therefore, the hydroxylation was assigned to the cyclobutyl ring even though fragments a and g were missing.

### Cyclopentyl fentanyl

All spectra of cyclopentyl fentanyl and metabolites are found in Supplemental Figure S3. The normetabolite (C7), the two hydroxylated normetabolites (C1, C2), the four mono- (C4, C6, C9) and dihydroxylated (C3) metabolites as well as the amide hydrolysis metabolite (C8) could be elucidated as described above.

The spectrum of metabolite C5 contains unmodified fragments b, c and e and the unsaturation indicated by the precursor mass cannot be located on the phenyl ring. Taken together, both the MSMS spectra and precursor mass are consistent with a carbonyl located on the cyclopentyl ring.

Similar to metabolite A7, C10 is likely an *N*-oxide positioned on the piperidine moiety nitrogen. This is supported by unmodified fragments b, g and f as well as a retention time greater than the parent.

### Cyclohexyl fentanyl

All spectra of cyclohexyl fentanyl and metabolites are found in Supplemental Figure S4. All metabolites except the monohydroxylated metabolite D10 could be elucidated as described above.

The spectra of D10 contain unmodified fragments b and g, as well as e + O, indicating a monohydroxylation on the piperidine ethyl moiety. Despite the late elution of the metabolite, fragment *m*/*z* 174 (formaldehyde loss from e + O) indicate a monohydroxylation rather than an *n*-oxide further supported by the lack of *m*/*z* 189 (observed for other *N*-oxides in this study).

### TMCPF

All spectra of TMCPF and metabolites are found in Supplemental Figure S5. The normetabolite (E9), the hydroxylated normetabolite (E1), the mono- (E5, E8) and dihydroxylated (E2) metabolites as well as the glucuronide (E3) were identified as described above.

The TMCP ring has been shown to rearrange into a ring opened form when subjected to heat or acid (Grigoryev et al. 2013). There is a possibility that some of the metabolites have rearranged rings or that they rearrange by the heat applied during ionization in the mass spectrometer. However, as the ring-opened product has the same mass as the product with the intact ring, it is not possible to distinguish them in this study. Therefore, all metabolites were drawn with intact rings. The fragment ion *m*/*z* 97.0623 in the spectra of E2 is more easily explained with a rearranged TMCP ring further indicating this possibility.

The spectra of metabolite E4 contains unmodified fragments b, d and e as well as g − H_2_. This is consistent with a monohydroxylated TMCP ring as well as a monohydroxylated *N*-phenyl ring. However, in all other spectra of metabolites with monohydroxylated TMCP rings (E1, E5 and E8) the g − H_2_ fragment is the base peak of the spectra and no other modifications on the *N*-phenyl ring were observed in this study. Based on this, it is possible that fragment g − H_2_ comes from a co-eluting compound and therefore a dihydroxylated TMCP ring cannot be ruled out.

The spectra of metabolites E6 and E7 are consistent with a carboxylic acid function on the TMCP ring as previously been shown for synthetic cannabinoid XLR-11 (Wohlfarth et al. 2013). Unmodified fragments b and e as well as fragment 281.1981, corresponding to the amide hydrolysis product, indicate that all modifications are on the TMCP ring. This is further supported by fragments g + O_2_ − H_2_ and a + O_2_ − H_2_. The fragment ion *m*/*z* 81, present in MSMS spectra for both E6 and E7, was formed by the loss of *m/z* 46 from the fragment ion *m/z* 127. As proposed in a previous study, the loss of CO_2_H_2_ to form *m/z* 81 most likely corresponds to either water and carbon monoxide or formic acid (Kotiaho et al. 2000).

Another pathway suggested by Wohlfarth et al. (2013) for XLR-11 is dioxidation followed by internal dehydration creating a novel 5 member ring containing an oxygen on the TMCP ring. Metabolite E10 appears to be consistent with this mechanism. Unmodified fragments b and e as well as fragment 281.2028, corresponding to the amide hydrolysis product, indicate that all modifications are on the TMCP ring. The spectra also contain fragment g + O − H_2_ (111.0795), indicating either the mentioned ring formation or aldehyde formation. However, due to the reactive nature of aldehyde metabolites (O’Brien et al. 2005) it is more likely that E10 is the ring formation metabolite.

### Major metabolic pathways

All metabolites were formed after 1 h and showed an increase in peak areas over time. Nevertheless, the rate of metabolite formation decreased after 3 h of incubation. Therefore, the 1 h samples were used when comparing the formation of different types of metabolites (Fig. 6).

Within this study, two major metabolic pathways could be identified: *N*-dealkylation (forming normetabolites) and oxidation at the alicyclic ring. *N*-dealkylation as biotransformation is a well-known pathway, described for fentanyl and fentanyl analog metabolism (Goromaru et al. 1984; Watanabe et al. 2017). Oxidation of the amide bound side chain as a major metabolic pathway is less common. However, it has been reported for furanyl fentanyl (Watanabe et al. 2017).

Figure 6 illustrates the observed shift from *N*-dealkylation as major pathway in favor of oxidation on the alicyclic ring as the ring size increases, potentially as the ring carbons become more accessible. Other pathways included oxidation on the piperidine moiety and amide hydrolysis. Apart from cyclopropyl fentanyl (4.8% of total area after 1 h) and cyclobutyl fentanyl (1.5%), no oxidation of the phenethyl substructure was observed. Similarly, no glucuronidated metabolites were observed for cyclobutyl, cyclopentyl and cyclohexyl fentanyl.

## Conclusion

Major metabolites of five alicyclic fentanyl analogs after incubation with human hepatocytes were described. All alicyclic fentanyl analogs were extensively modified by major metabolic pathways such as *N*-dealkylation resulting in formation of normetabolites and oxidation reactions of the alicyclic ring. Interestingly, a correlation of the number of carbons in the alicyclic ring with *N*-dealkylation was found. This indicates that the significance of *N*-dealkylation as a major biotransformation decreased in favor of alicyclic ring oxidation.

## Electronic supplementary material

Below is the link to the electronic supplementary material.

Supplementary material 1 (PDF 2478 KB)
