# Supplementary material for: Correlations between metabolism and structural elements of the alicyclic fentanyl analogs cyclopropyl fentanyl, cyclobutyl fentanyl, cyclopentyl fentanyl, cyclohexyl fentanyl and 2,2,3,3-tetramethylcyclopropyl fentanyl studied by human hepatocytes and LC-QTOF-MS
Source: Arch Toxicol. 2018 Oct 25;93(1):95–106. doi: 10.1007/s00204-018-2330-9 (PMC6342890; doi:10.1007/s00204-018-2330-9)
Supplement: Supplementary file 1 — Supplementary material 1 (PDF 2478 KB) [file 204_2018_2330_MOESM1_ESM.pdf]

Correlations between metabolism and structural elements of alicyclic fentanyl analogs cyclopropyl fentanyl, cyclobutyl fentanyl, cyclopentyl fentanyl, cyclohexyl fentanyl and 2,2,3,3-tetramethylcyclopropyl fentanyl studied by human hepatocytes and LC-QTOF-MS

Supplementary Material

Anna Åstrand[1], Amanda Töreskog[2], Shimpei Watanabe[2], Robert Kronstrand[1,2], Henrik Gréen[1,2], Svante Vikingsson[1,2]

1 Department of Medical and Health Sciences, Linköping University, Linköping, Sweden

2 Department of Forensic Genetics and Forensic Toxicology, National Board of Forensic Medicine, Linköping, Sweden.

Supplementary table S1 *– Identified metabolites of cyclopropyl fentanyl (A) with assigned ID, biotransformation, retention time (RT), accurate mass of protonated metabolite, mass error, peak areas and diagnostic fragment ions.*

						Peak area (counts)				
ID	Biotransformation	Formula	RT (min)	m/z	mass error (ppm)	0h	1h	3h	5h	Diagnostic fragment ions (m/z)
A1	N-dealkylation	C15 H20 N2 O	7,86	245,1648	-0,10	98417<br>ND	6 964 533<br>6 309 717	10 714 175<br>11 810 619	12 864 182<br>13 485 950	69.0333, 84.0806, 162.0912, 177.1385
A2	Dihydrodiol / Ring opening + dihydroxylation	C23 H30 N2 O3	8,25	383,2326	-0,95	ND<br>ND	98 229<br>93 668	142 389<br>149 102	151 856<br>154 359	69.0331, 146.0949, 189.1380, 204.1378
A3	Monohydroxylation at the phenethyl substructure + Glucuronidation	C29 H36 N2 O8	8,33	541.2541	-1.11	ND<br>ND	ND<br>ND	52 376<br>55 913	72 943<br>83 653	69.0311, 121.0640, 204.1383
A4	Monohydroxylation at the phenethyl substructure	C23 H28 N2 O2	10,05	365,2223	-0,38	ND<br>ND	361 645<br>211 927	345 469<br>493 754	557 492<br>556 838	69.0330, 121.0647, 204.1376
A5	Methylation + Dihydroxylation at the phenethyl substructure	C24 H30 N2 O3	10,35	395,2311	-5,55	ND<br>ND	ND<br>ND	38 680<br>46 857	61 375<br>56 039	69.0327, 84.0802, 151.0751, 180.1014, 234.1446
A6	Monohydroxylation at the piperidine ethyl moiety	C23 H28 N2 O2	10,79	365,2221	-0,61	ND<br>ND	1 119 452<br>587 657	1 151 101<br>1 923 875	2 330 753<br>2 325 024	69.0334, 105.0704, 132.0808,146.0959, 174.1282, 204.1373, 228.1371
A	Cyclopropyl fentanyl	C23 H28 N2 O	11,76	349,2275	0,14	29 374 542<br>23 738 129	28 035 785<br>23 482 084	22 476 235<br>25 944 138	25 296 342<br>24 526 800	69.0333, 105.0698, 132.0806, 134.0963, 188.1434, 228.1380
A7	N-oxide at the piperidine moiety	C23 H28 N2 O2	12,97	365,2223	-0,26	ND<br>ND	253 154<br>214 042	317 002<br>399 872	383 815<br>443 134	69.0328, 105.0692, 146.0971, 189.1387, 228.1370, 257.1662

ND: not detected

***Supplementary table S2** – Identified metabolites of cyclobutyl fentanyl (B) with assigned ID, biotransformation, retention time (RT), accurate mass of protonated metabolite, mass error, peak areas and diagnostic fragment ions.*

						Peak areas (counts)				
ID	Biotransformation	Formula	RT (min)	m/z	Mass error (ppm)	0h1	1h	3h1	5h1	Diagnostic fragment ions (m/z)
B1	N-dealkylation + Monohydroxylation at the alicyclic ring	C16 H22 N2 O2	5,33	275,1751	-0,97	ND<br>ND	100 198<br>102 751	158 993<br>168 152	295 678<br>285 565	84.0810, 99.0448, 192.0973
B2	Monohydroxylation at the alicyclic ring	C24 H30 N2 O2	9,26	379,2373	-1,7	ND<br>ND	259 005<br>359 146	553 847<br>796 524	768 761<br>661 862	99.0426, 105.0692, 132.0794, 188.1429
B3	N-dealkylation	C16 H22 N2 O	9,33	259,1833	8,11	81 242<br>82 016	8 148 478<br>9 282 392	9 745 780<br>11 303 860	12 187 512<br>11 685 789	55.0539, 84.0805, 177.1378
B4	Monohydroxylation at the alicyclic ring	C24 H30 N2 O2	9,54	379,2393	2,4	ND<br>ND	3 032 710<br>4 190 723	6 403 987<br>8 110 019	7 861 096<br>7 366 250	71.0482, 105.0696, 134.0957, 188.1427
B5	Dihydroxylation at the alicyclic ring	C24 H30 N2 O3	9,66	395,2328	-0,54	ND<br>ND	67 832<br>74 991	193 314<br>218 625	415 299<br>408 228	87.0441, 105.0693, 134.0949, 188.1433
B6	Amide hydrolysis + Monohydroxylation at the piperidine ethyl moiety	C19 H24 N2 O	9,79	297,1956	-1,53	ND<br>ND	40 876<br>53 138	404 354<br>487 311	625 070<br>562 540	105.0688, 134.0961, 146.0938, 174.1262, 186.1269, 204.1364
B7	Monohydroxylation at the alicyclic ring	C24 H30 N2 O2	10,36	379,2373	-1,63	ND<br>ND	554 629<br>922 357	1 108 224<br>1 807 744	1 556 139<br>1 367 102	105.0692, 134.0952, 146.0940, 188.1429, 281.1993
B8	Amide hydrolysis	C19 H24 N2	11,06	281,2019	1,83	ND<br>ND	1 227 030<br>1 693 103	7 035 964<br>8 000 160	7 542 944<br>6 957 798	105.0694, 134.0956, 188.1429
B9	Monohydroxylation at the phenethyl substructure	C24 H30 N2 O2	11,34	379,2375	-1,81	ND<br>ND	184 560<br>296 428	262 744<br>422 025	264 132<br>230 562	55.0531, 84.0510, 121.0635, 204.1379
B10	Monohydroxylation at the piperidine ethyl moiety	C24 H30 N2 O2	12,02	379,2372	-1,83	ND<br>ND	797 826<br>1 352 217	1 457 518<br>2 420 694	1 507 579<br>1 208 972	55.0538, 105.0694, 132.0803, 174.1270 204.1375, 242.1526
B	Cyclobutyl fentanyl	C24 H30 N2 O	12,95	363,2479	13,33	20 299 112<br>20 031 315	19 731 279<br>21 676 089	19 147 596<br>20 824 770	17 286 082<br>15 893 004	55.0539, 105.0693, 132.0804, 134.0960,188.1431,242.1539

ND: not detected

***Supplementary table S3** – Identified metabolites of cyclopentyl fentanyl (C) with assigned ID, biotransformation, retention time (RT), accurate mass of protonated metabolite, mass error, peak areas and diagnostic fragment ions.*

ID	Biotransformations	Formula	RT (min)	m/z	Mass error (ppm)	Peak areas (counts) 0h	1h	3h	5h	Diagnostic fragment ions (m/z)
C1	N-dealkylation + Monohydroxylation at the alicyclic ring	C17 H24 N2 O2	2,21	289,191	-0,18	ND<br>ND	187893<br>181975	344718<br>359419	463206<br>461434	67.0543, 84.0807, 113.0576, 177.1374, 206.1148
C2	N-dealkylation + Monohydroxylation at the alicyclic ring	C17 H24 N2 O2	2,98	289,1915	1,33	ND<br>ND	977 005<br>969 625	2 669 733<br>2 815 602	3 402 846<br>3 574 216	67.0539, 84.0807, 113.0597, 177.1384, 188.1067
C3	Dihydroxylation at the piperidine ethyl moiety and the alicyclic ring	C25 H32 N2 O3	4,84	409,2483	-0,57	ND<br>ND	558 425<br>580 738	1 376 120<br>1 460 377	1 561 480<br>1 582 240	67.0540, 105.0698, 132.0801, 174.1278, 204.1378
C4	Monohydroxylation at the alicyclic ring	C25 H32 N2 O2	4,86	393,2543	0,9	ND<br>ND	3 518 963<br>4 116 230	5 467 836<br>5 549 603	5 395 439<br>5 104 506	67.0541, 85.0648, 105.0698, 134.0961, 188.1434
C5	Ketone formation at the alicyclic ring	C25 H30 N2 O2	5,41	391,2382	0,4	ND<br>ND	1 216 659<br>1 464 012	2 587 879<br>2 606 457	2 801 909<br>2 846 724	105.0698, 132.0807, 188.1435
C6	Monohydroxylation at the alicyclic ring	C25 H32 N2 O2	5,57	393,2576	10,14	ND<br>ND	10 217 759<br>11 341 192	15 019 642<br>15 062 712	14 178 910<br>14 113 836	67.0542, 105.0700, 134.0962, 188.1438
C7	N-dealkylation	C17 H24 N2 O	5,58	273,2003	15,38	372 990<br>253 982	11 053 075<br>11 923 548	13 204 788<br>13 073 264	13 342 508<br>13 078 561	69.0702, 84.0810, 97.0649, 177.1388
C8	Amide hydrolysis	C19 H24 N2	6,09	281,2014	0,72	ND<br>ND	934 022<br>1 648 558	2 673 558<br>1 300 280	2 183 851<br>1 870 090	105.0698, 134.0966, 188.1436
C9	Monohydroxylation at the piperidine ethyl moiety	C25 H32 N2 O2	7,57	393,2534	-0,64	ND<br>ND	3 446 829<br>5 253 248	4 142 971<br>3 037 914	2 326 838<br>2 083 848	69.0700, 105.0696, 132.0810, 174.1278, 204.1389
C	Cyclopentyl fentanyl	C25 H32 N2 O	8,29	377,2631	11,57	25 804 612<br>23 496 421	22 183 498<br>25 608 452	20 149 501<br>17 428 815	15 004 716<br>15 089 766	69.0700, 105.0699, 132.0809, 134.0963, 188.1436, 256.1696
C10	N-oxide at the piperidine moiety	C25 H32 N2 O2	9,17	393,2539	0,51	ND<br>ND	496 224<br>596 146	709 267<br>677 510	572 915<br>535 487	69.0697, 105.0697, 132.0802, 146.0965, 186.1279, 189.1382, 256.1693

ND: not detected

Supplementary table S4 *– Identified metabolites of cyclohexyl fentanyl (D) with assigned ID, biotransformation, retention time (RT), accurate mass of protonated metabolite, mass error, peak areas and diagnostic fragment ions.*

						Peak areas (count)				
ID	Biotransformation	Formula	RT (min)	m/z	Mass error (ppm)	0h	1h	3h	5h	Diagnostic fragment ions (m/z)
D1	Dihydroxylation at the piperidine ethyl moiety and the alicyclic ring	C26 H34 N2 O3	4,13	423,2642	-0,43	ND<br>ND	161 775<br>179 711	236 861<br>304 193	351 007<br>353 075	81.0699, 105.0685, 132.0792, 174.1267, 204.1407
D2	Amide hydrolysis + Monohydroxylation at the phenethyl substructure	C19 H24 N2 O	4,51	297,1961	-0,29	ND<br>ND	ND<br>ND	82 684<br>168 642	201 502<br>212 284	121.0646, 150.09113, 204.1372
D3	Monohydroxylation at the alicyclic ring	C26 H34 N2 O2	4,95	407,2708	2,59	ND<br>ND	5 778 945<br>6 979 425	6 700 939<br>9 393 321	9 170 541<br>9 431 952	81.0696, 105.0696, 134.0958, 188.1428, 286.1793
D4	Amide hydrolysis + Monohydroxylation at the piperidine ethyl moiety	C19 H24 N2 O	5,13	297,1958	-0,92	ND<br>ND	205 771<br>263 152	587 536<br>1 318 890	1 307 325<br>1 315 846	105.0692, 134.0854,174.1277, 204.1375
D5	Monohydroxylation at the alicyclic ring	C26 H34 N2 O2	5,42	407,2692	-0,31	ND<br>ND	151 444<br>198 613	167 641<br>275 122	234 795<br>244 330	81.0687,105.0693, 134.0946, 188.1422, 286.1795
D6	Monohydroxylation at the alicyclic ring	C26 H34 N2 O2	5,58	407,2697	0,45	ND<br>ND	3 793 989<br>5 131 395	3 488 962<br>4 533 477	5 543 882<br>5 730 021	81.0693, 105.0695, 134.0956, 188.1438, 286.1744
D7	Monohydroxylation at the alicyclic ring	C26 H34 N2 O2	5,82	407,2691	-0,46	ND<br>ND	1 868 313<br>2 551 175	1 861 171<br>3 082 014	2 820 635<br>2 841 722	81.0697, 105.0690, 134.0955, 188.1438, 286.1744
D8	Amide hydrolysis	C19 H24 N2		281,2046	11,99	ND<br>ND	6 232 759<br>7 748 393	9 877 668<br>15 442 502	13 627 358<br>13 731 121	105.0694, 134.0961,188.1431
D9	N-dealkylation	C18 H26 N2 O	6,30	287,2137	5,14	78 814<br>64 133	6 577 247<br>7 174 952	7 844 530<br>8 245 851	8 800 972<br>8 973 538	83.0848, 84.0802, 177.1386
D10	Monohydroxylation at the piperidine ethyl moiety	C26 H34 N2 O2	8,23	407,2695	0,37	ND<br>ND	671 054<br>1 067 886	286 481<br>678 106	271 822<br>263 049	83.0848, 105.0698, 132.0796, 174.1244, 204.1386, 270.1862
D	Cyclohexyl fentanyl	C26 H34 N2 O	8,94	391,2764	3,76	24 932 926<br>26 358 131	27 435 850<br>31 212 866	14 843 790<br>26 795 006	12 852 998<br>12 663 853	83.0853, 105.0694, 134.0958, 188.1433, 270.1837

ND: not detected

Supplementary table S5 *– Identified metabolites of 2,2,3,3-tetramethylcyclopropyl fentanyl (TMCPF) (E) with assigned ID, biotransformation, retention time (RT), accurate mass of protonated metabolite, mass error, peak areas and diagnostic fragment ions.*

ID	Biotransformation	Formula	RT (min)	m/z	mass error (ppm)	Peak area (counts) 0h	1h	3h	5h	Diagnostic fragments ions (m/z)
E1	N-dealkylation + Monohydroxylation at the alicyclic ring	C19 H28 N2 O2	3,47	317,2219	-1,4	ND<br>ND	797 000<br>681 000	1 470 000<br>1 280 000	1 680 000<br>1 950 000	84.0800, 95.0852, 123.0802, 141.0919
E2	Dihydroxylation at the alicyclic ring	C27 H36 N2 O3	4,69	437,2794	-0,96	ND<br>ND	1 060 000<br>1 220 000	1 630 000<br>2 810 000	2 530 000<br>2 590 000	93.0700, 105.0692, 134.0945, 188.1432, 281.1997
E3	Monohydroxylation at the alicyclic ring + Glucuronidation	C33 H44 N2 O8	4,95	597,3167	-0,42	ND<br>ND	864 000<br>754 000	1 710 000<br>1 080 000	1 440 000<br>1 690 000	95.0850, 105.0691, 123.0804, 141.0910, 188.1435, 421.2837
E4	Dihydroxylation at the alicyclic ring	C27 H36 N2 O3	5,41	437,2797	-0,49	ND<br>ND	317 000<br>359 000	454 000<br>903 000	768 000<br>759 000	95.0858, 105.0694, 134.0958, 188.1439
E5	Monohydroxylation at the alicyclic ring	C27 H36 N2 O2	6,42	421,2847	-0,62	ND<br>ND	10 500 000<br>12 300 000	8 980 000<br>16 700 000	11 300 000<br>11 200 000	95.0852, 105.0691, 123.0791, 141.0910, 188.1429
E6	Carboxylation at the alicyclic ring	C27 H34 N2 O3	6,45	435,2641	-0,44	ND<br>ND	4 870 000<br>4 230 000	8 300 000<br>6 930 000	9 160 000<br>10 200 000	81.0697, 105.0694, 127.0752, 155.0696, 188.1429, 281.1981
E7	Carboxylation at the alicyclic ring	C27 H34 N2 O3	6,88	435,2643	-0,21	ND<br>ND	521 000<br>488 000	1 060 000<br>966 000	1 250 000<br>1 400 000	81.0689, 105.0694, 127.0752, 155.0696, 188.1429, 281.1981
E8	Monohydroxylation at the alicyclic ring	C27 H36 N2 O2	7,42	421,2846	-0,74	ND<br>ND	2 650 000<br>3 340 000	1 590 000<br>4 700 000	2 180 000<br>2 070 000	95.0848, 105.0695, 134.0952, 141.0921, 188.1434
E9	N-dealkylation	C19 H28 N2 O	7,65	301,2272	-0,69	168 000<br>ND	3 920 000<br>4 270 000	4 350 000<br>6 670 000	5 480 000<br>5 580 000	84.0803, 97.1007, 125.0959
E10	Dihydroxylation + internal dehydration at the alicyclic ring	C27 H34 N2 O2	7,82	419,2691	-0,69	ND<br>ND	765 000<br>1 020 000	644 000<br>2 130 000	1 200 000<br>1 010 000	105.0695, 111.0795, 134.0948, 188.1428, 281.2028
E	Tetramethylcyclopropyl fentanyl	C27 H36 N2 O	10,31	405,2901	0,01	38 700 000<br>24 500 000	19 500 000<br>25 400 000	6 500 000<br>18 600 000	9 940 000<br>11 900 000	97.1010, 105.0693, 125.0960, 134.0959, 188.1431

ND: not detected

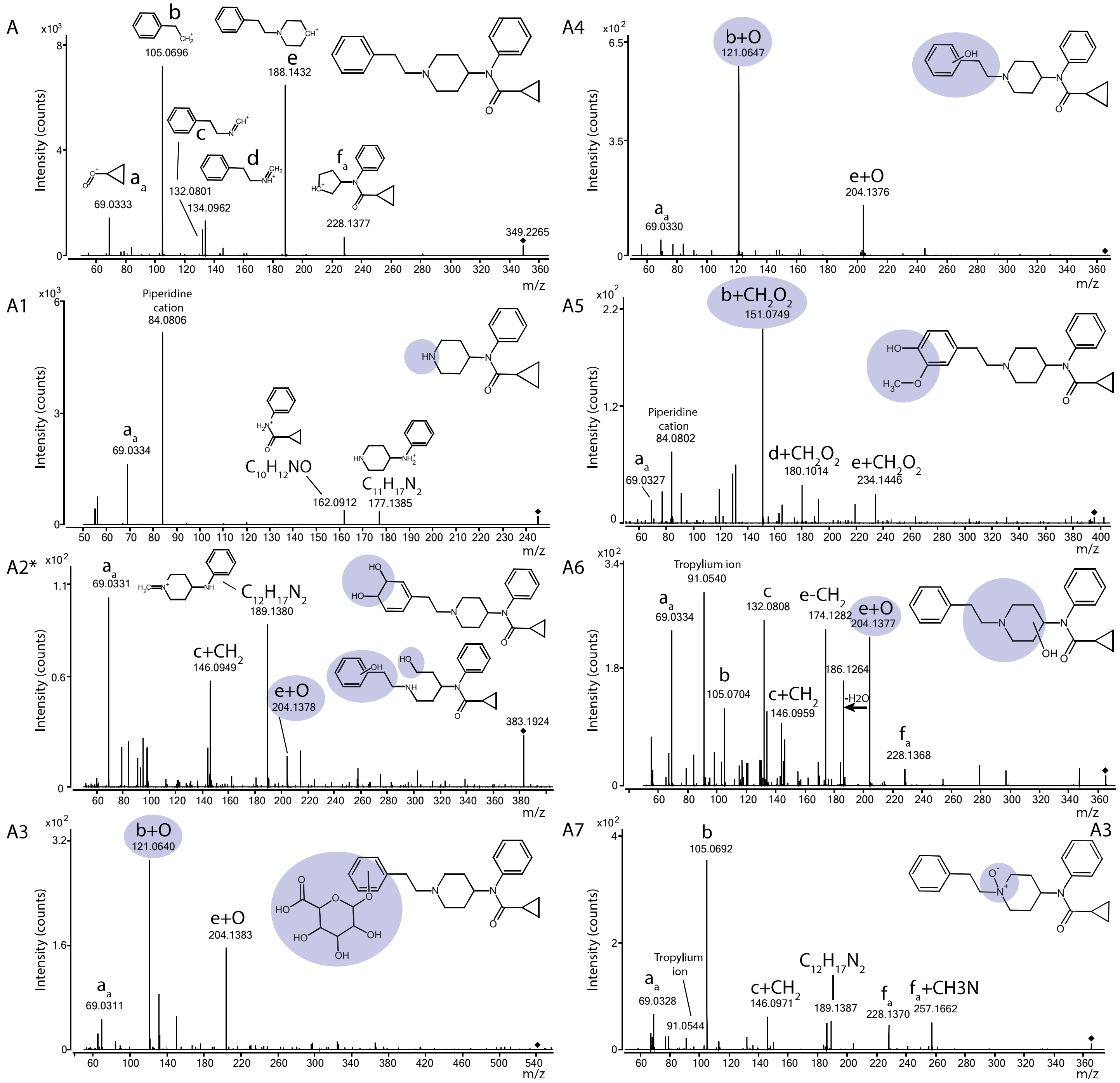

Supplementary figure S1- MSMS-spectra of cyclopropylfentanyl (A) and detected cyclopropylfentanyl metabolites (A1-A7). Suggested fragmentation pattern for cyclopropylfentanyl is depicted in the top left panel (A) and the fragment ions are labeled a_a, b, c, d, e and f_a. The fragment ions displayed in the metabolites MSMS-spectra are labeled using the same letters plus or minus any structural modifications. The structure of each metabolite is shown next to the corresponding MSMS-spectrum with modifications highlighted in blue. Key fragment ions used for structure elucidation are also highlighted in blue. * Two possible strucutres are shown for A2.

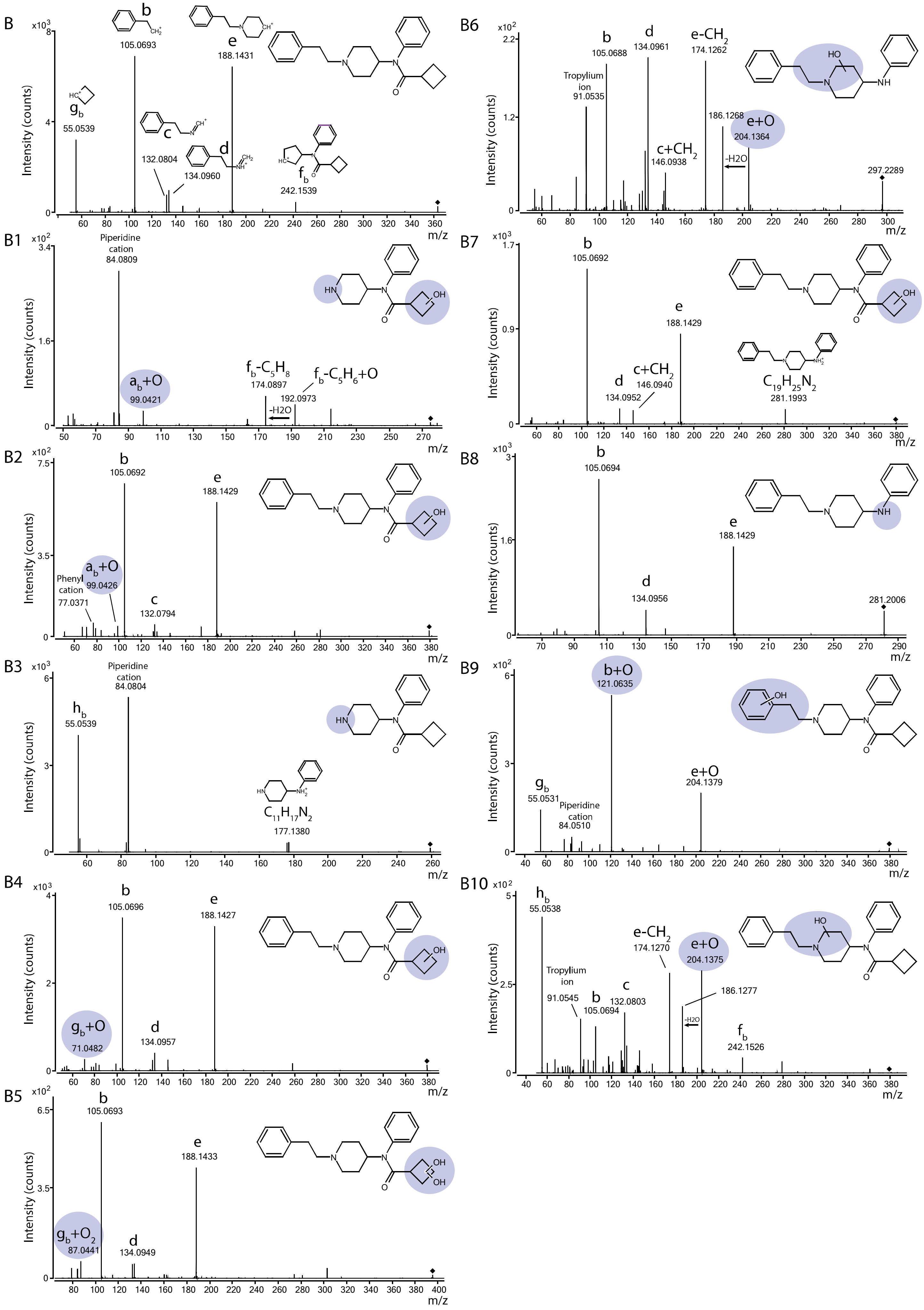

Supplementary figure S2- MSMS-spectra of cyclobutylfentanyl (B) and detected cyclobutylfentanyl metabolites (B1-B10). Suggested fragmentation pattern for cyclobutylfentanyl is depicted in the top left panel (B) and the fragment ions are labeled a_b, b, c, d, e , f_b and g_b. The fragment ions displayed in the metabolites MSMS-spectra are labeled using the same letters plus or minus any structural modifications. The structure of each metabolite is shown next to the corresponding MSMS-spectrum with modifications highlighted in blue. Key fragment ions used for structure elucidation are also highlighted in blue

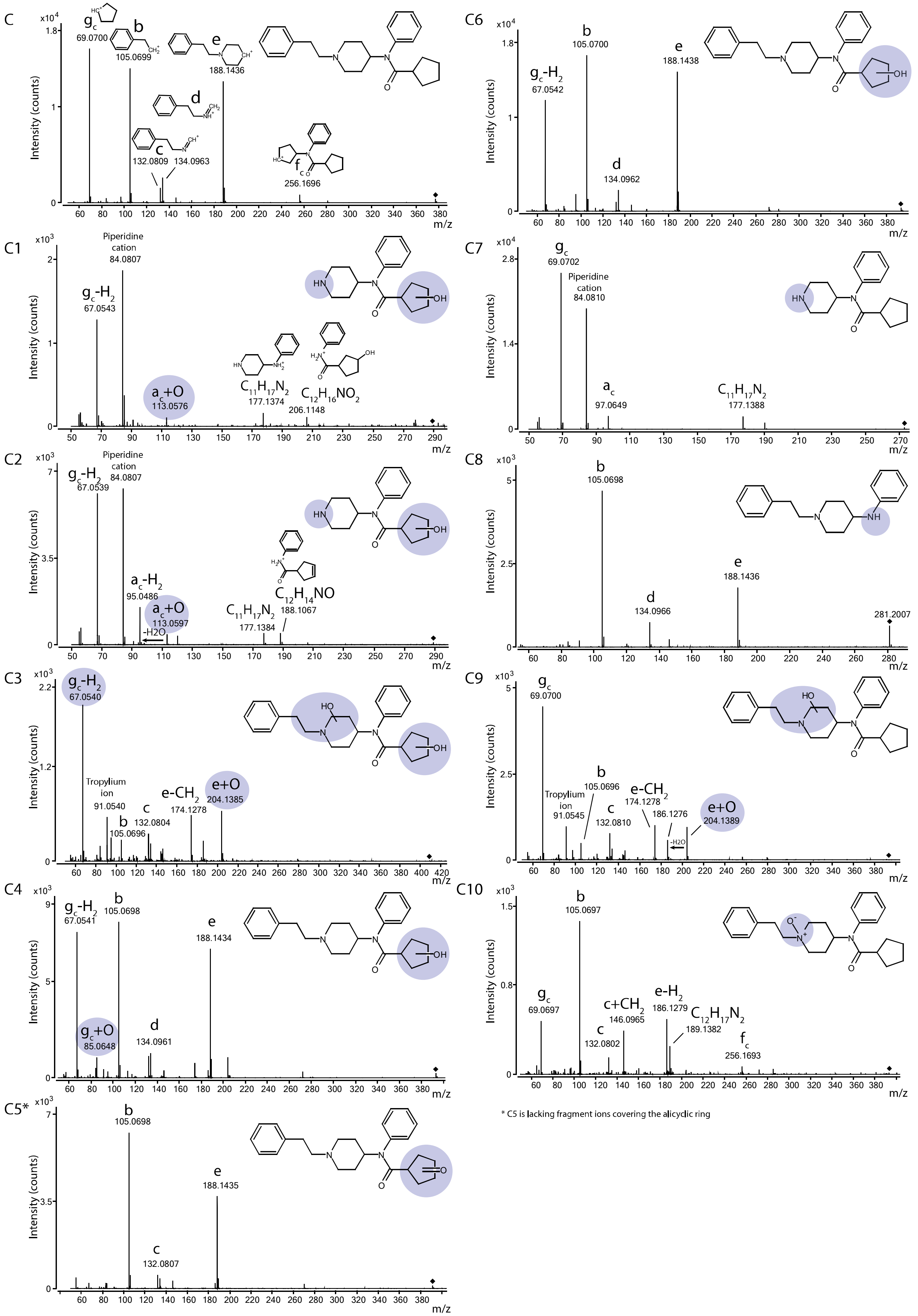

Supplementary figure S3- MSMS-spectra of cyclopentylfentanyl (C) and detected cyclopentylfentanyl metabolites (C1-C10). Suggested fragmentation pattern for cyclopentylfentanyl is depicted in the top left panel (C) and the fragment ions are labeled a_c, b, c, d, e , f_c and g_c. The fragment ions displayed in the metabolites MSMS-spectra are labeled using the same letters plus or minus any structural modifications. The structure of each metabolite is shown next to the corresponding MSMS-spectrum with modifications highlighted in blue. Key fragment ions used for structure elucidation are also highlighted in blue

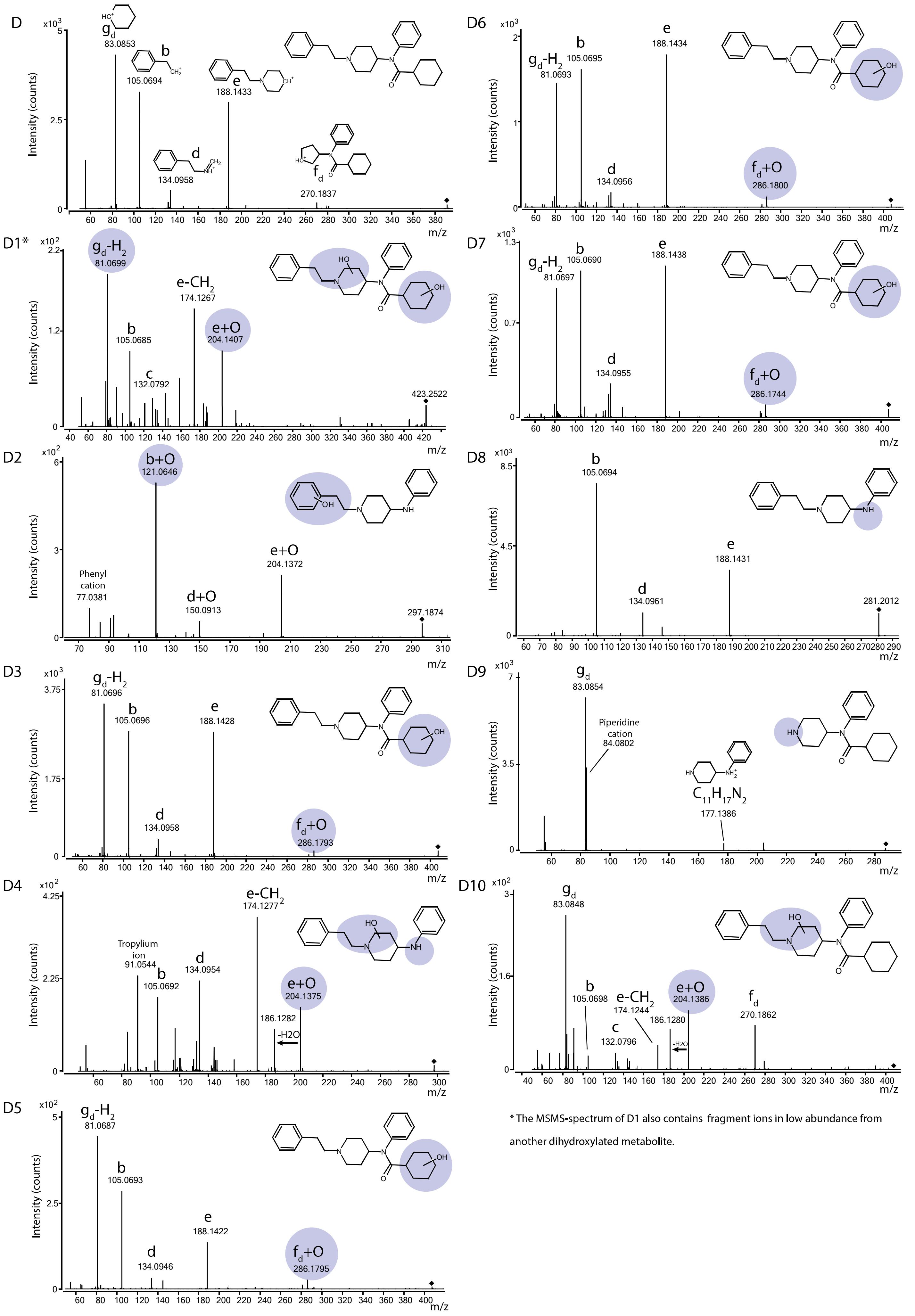

* The MSMS-spectrum of D1 also contains fragment ions in low abundance from another dihydroxylated metabolite.

Supplementary figure S4- MSMS-spectra of cyclohexylfentanyl (D) and detected cyclohexylfentanyl metabolites (D1-D10). Suggested fragmentation pattern for cyclohexylfentanyl is depicted in the top left panel (D) and the fragment ions are labeled a_d, b, c, d, e , f_d and g_d. The fragment ions displayed in the metabolites MSMS-spectra are labeled using the same letters plus or minus any structural modifications. The structure of each metabolite is shown next to the corresponding MSMS-spectrum with modifications highlighted in blue. Key fragment ions used for structure elucidation are also highlighted in blue

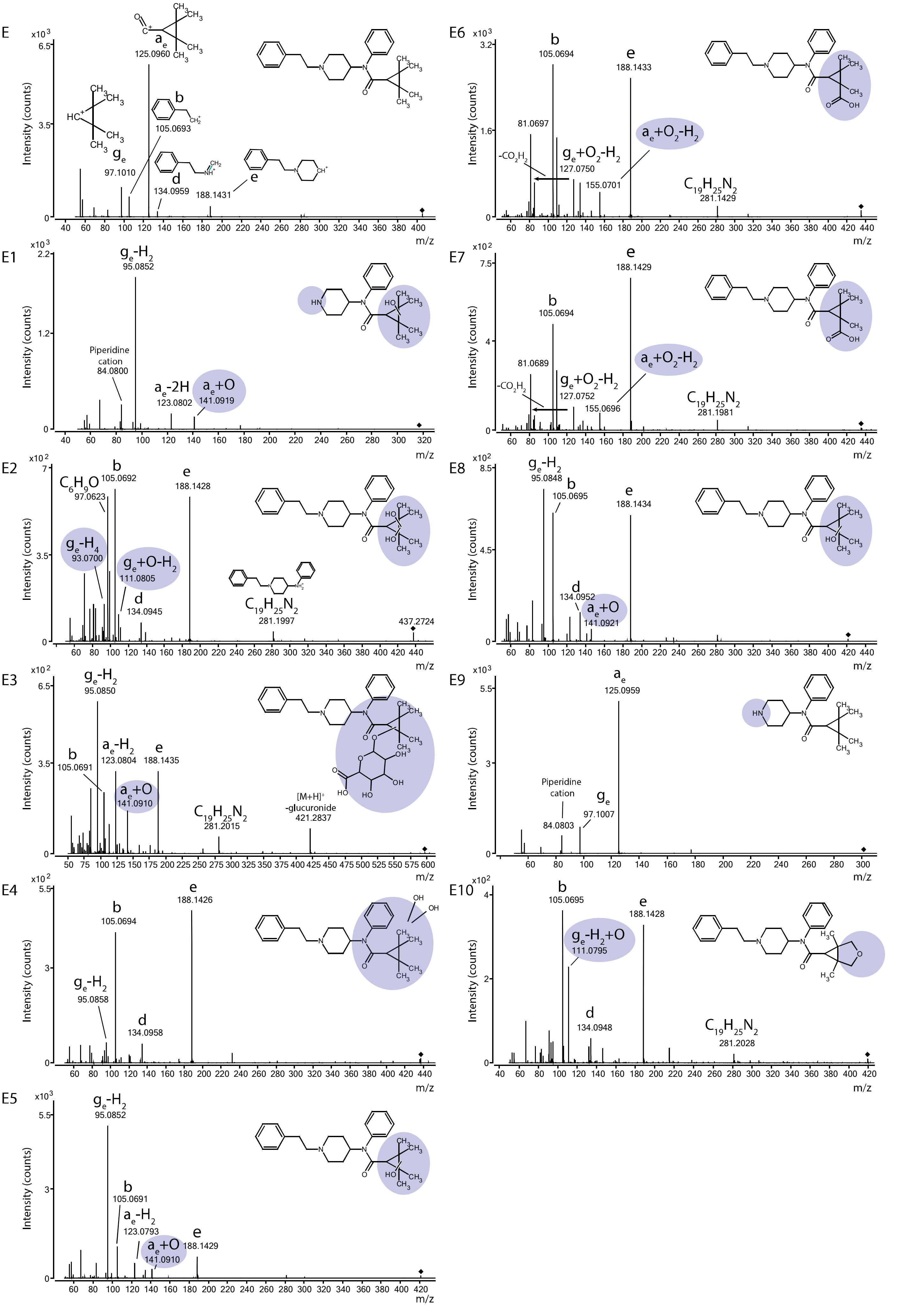

Supplementary figure S5 - MSMS-spectra of 2,2,3,3-tetramethylcyclopropylfentanyl (TMCPF) (E) and detected TMCPF metabolites (E1-E10). Suggested fragmentation pattern for TMCPF is depicted in the top left panel (E) and the fragment ions are labeled a_e, b, d, e, and g_e. The fragment ions displayed in the metabolites MSMS-spectra are labeled using the same letters plus or minus any structural modifications. The structure of each metabolite is shown next to the corresponding MSMS-spectrum with modifications highlighted in blue. Key fragment ions used for structure elucidation are also highlighted in blue.
